# Plasma neurofilament light chain: A potential prognostic biomarker of dementia in adult Down syndrome patients

**DOI:** 10.1371/journal.pone.0211575

**Published:** 2019-04-05

**Authors:** Makiko Shinomoto, Takashi Kasai, Harutsugu Tatebe, Masaki Kondo, Takuma Ohmichi, Masafumi Morimoto, Tomohiro Chiyonobu, Naoto Terada, David Allsop, Isao Yokota, Toshiki Mizuno, Takahiko Tokuda

**Affiliations:** 1 Department of Neurology, Kyoto Prefectural University of Medicine, Graduate School of Medical Science, Kyoto, Japan; 2 Department of Zaitaku (Homecare) Medicine, Kyoto Prefectural University of Medicine, Kyoto, Japan; 3 Department of Pediatrics, Kyoto Prefectural University of Medicine, Graduate School of Medical Science, Kyoto, Japan; 4 Department of Pediatrics, North Medical Center, Kyoto Prefectural University of Medicine, Kyoto, Japan; 5 Hananoki Medical Welfare Center, Kyoto, Japan; 6 Division of Biomedical and Life Sciences, Faculty of Health and Medicine, Lancaster University, Lancaster, United Kingdom; 7 Department of Biostatistics, Kyoto Prefectural University of Medicine, Kyoto, Japan; 8 Department of Molecular Pathobiology of Brain Diseases, Kyoto Prefectural University of Medicine, Kyoto, Japan; Nathan S Kline Institute, UNITED STATES

## Abstract

People with Down syndrome (DS) are at high risk of developing Alzheimer disease (AD) with aging. The diagnosis and treatment trials are hampered by a lack of reliable blood biomarkers. Plasma neurofilament light chain (NfL) is one of the established biomarkers of AD, suggesting that it may be useful as an indicator of dementia in DS patients. The aims of this study were: 1) to examine whether plasma levels of NfL in DS patients are correlated with decreased adaptive behavior scores one year after sample collection, and 2) to compare plasma levels of NfL in adults with DS and an age-matched healthy control population. In this study, plasma levels of NfL in 24 patients with DS and 24 control participants were measured by the single-molecule immunoarray (Simoa) method. We observed significantly increased plasma NfL levels in the DS compared with the control group. There was a significant correlation between age and levels of plasma NfL in both groups. This age-dependent elevation was steeper in the DS compared with the control group. Moreover, elevated plasma NfL was associated with decreased adaptive behavior scores one year later, after age-adjustment. Previously reported blood-based biomarkers available in Simoa for DS, plasma total tau and phosphorylated tau, were not significantly correlated with the annual decrement of adaptive behavior scores after age-adjustment. These results suggest that plasma NfL has the potential to serve as an objective biomarker to predict dementia in adult DS patients.

## Introduction

Over the last few decades, there have been significant improvements both in the live birth prevalence and survival rates of people with DS because of better health care in developed countries. Consequently, their average life expectancy has markedly improved and now exceeds 50 years [1, 2]. This general aging of the DS population has created an important issue for neurologists because many of them over 40 years old are at risk of developing dementia. Pathological brain changes of aged individuals with DS are almost identical to those of patients with Alzheimer’s disease (AD), consisting of both senile plaques and neurofibrillary tangles composed of amyloid β (Aβ) and phosphorylated tau, respectively [3]. The causative mechanism is considered to involve the triplication of *APP* on chromosome 21, leading to a brain pathology indicative of AD, as well as overexpressions of *Dyrk1A* and *RCAN1* also located on chromosome 21, which are both involved in tau hyperphosphorylation [4].

Diagnosing dementia in patients with DS, particularly in the early stage, is difficult due to their markedly variable baseline cognitive abilities as well as heterogeneity of dementia symptoms [5]. Meanwhile, there is an urgent need to develop diagnostic tools for early dementia of DS because disease-modifying therapies under development for AD, aiming at the reduction of Aβ and/or tau aggregation, could also be effective for the prevention of dementia in DS patients.

Considering the similarity between dementia in DS and AD patients, a logical approach for the objective detection of dementia in DS patients would be to employ biomarkers used for the diagnosis of AD. In fact, Aβ and tau deposition detected by positron emission tomography (PET), and the levels of these molecules in cerebrospinal fluid (CSF), were reported to have diagnostic value for dementia in DS patients [6–8]. Owing to recent advances in immune-complex-based technologies, including single-molecular array (Simoa), these molecules can be detected even in blood samples and blood markers would be more cost-effective and less invasive than PET or CSF-based ones [9] [10] [11] [12].

Neurofilament light chain (NfL) is one of the scaffolding cytoskeleton proteins of myelinated subcortical axons. NfL, which can now be reliably measured even in sera and plasma using Simoa, is an indicator of axonal injury due to several neurological diseases, including neurodegenerative ones. In contrast to Aβ and tau, altered levels of NfL do not specifically indicate the presence of AD pathology. However, this lack of specificity is less of an issue because most cases of dementia in DS patients are thought to be based on AD pathology. To our knowledge, only two previous studies have reported an age-dependent elevation of plasma levels of NfL in DS patients [13] [14]. However, it remains unclear whether increased plasma NfL can be used to predict future cognitive decline, based on longitudinal studies. Furthermore, age-dependent elevation of plasma NfL was also observed in healthy individuals [15] [16] [17]; therefore, the question of when abnormal elevation of plasma NfL starts in DS patients remains to be answered by comparing their datasets with those of control individuals. The aims of this study were: 1) to compare plasma levels of NfL in adults with DS and an age-matched healthy control population, and 2) to examine whether plasma levels of NfL in DS patients are correlated with decreased adaptive behavior scores one year after sample collection.

## Materials and methods

### Study design, ethics statement, and subject recruitment

All study subjects provided written informed consent before participation and the study protocols were approved by the University Ethics Committee (RBMR-C-1226 for participants with DS and ERB-G-12 for controls, Kyoto Prefectural University of Medicine, Kyoto, Japan). Informed consent from patients in the DS group was obtained when possible and from the nearest relative. Study procedures were designed and performed in accordance with the Declaration of Helsinki. We enrolled 24 adult patients with DS (DS group) from the registration for DS in Kyoto Prefectural University of Medicine and Hananoki Medical Welfare Center, from February 2013 to January 2018. Age-matched healthy volunteers or patients presenting with neither neurological symptoms nor head injury within three months prior to sample collection were enrolled as the control group. Individuals with any known chromosomal abnormality, neurodegenerative disease, or a family history of AD involving first-degree relatives, were excluded from this group. Plasma from 24 individuals in the control group was obtained from another registration of Kyoto Prefectural University of Medicine during the aforementioned period. Plasma samples were obtained via venous puncture: a total of 8 mL of blood was collected in EDTA-containing tubes. After collection, plasma was separated by centrifugation for 10 min at 3,000 rpm and placed in polypropylene vials. Fresh samples obtained from the enrolled subjects were immediately stored at -80°C until analysis.

Information on the following comorbid diseases and conditions were retrospectively collected from clinical charts of the DS group at sample collection: treatment or diagnosis of sleep apnea, epilepsy, hyperuricemia, hypothyroidism, and head injury within the last three months.

The adaptive behavior score in the DS group was estimated as the ‘social age’ using the social maturity scale revised (S-M) (S-M scale)(Nihonbuknasha, Tokyo), which is an adaptive behavior scale developed for Japanese based on the Vineland Social Maturity Scale [18]. The test consists of 130 yes/no questions evaluating self-help (31 questions), locomotion (18 questions), occupation (19 questions), communication (23 questions), socialization (21 questions), and self-direction (18 questions). Social ages were calculated from raw scores in the conversion table based on data for typically developing Japanese children. The initial examination was performed within 3 months after blood collection. We annually evaluated the social age of 13 participants with DS. As a few participants with stable cognition received the assessment behind schedule, follow-up tests were done from 12 to 18 months after the initial examination. The same caregiver completed follow-up assessments. Annual decrement of the social age (referred as %ΔSocial Age) was calculated using the formula: (social age the first time—social age the second time) x 100 / (social age the first time). Patients with DS were also systematically screened using Dementia Screening Questionnaires for Individuals with Intellectual Disabilities [19] (DSQIID, the Japanese edition was obtained from the following website: http://www.nozomi.go.jp/investigation/pdf/report/04/01.pdf).We previously reported plasma levels of total tau (t-tau) [11] and phosphorylated tau (p-tau) [10], DSQIID, and the adaptive behavior score of the 19 participants with DS elsewhere.

### Measurement of NfL

The plasma NfL concentration was measured with reagents from a single lot using the Simoa NF-light assay (a digital sandwich immunoassay employing antibodies directed against the rod domain of NF-L) on an HD-1 Simoa analyzer according to the protocol issued by the manufacturer (Quanterix, Lexington, MA, USA). All samples were analyzed in duplicate on one occasion. All cases and controls were evenly distributed on examination. The plasma NfL was measured only in samples collected at the time of the initial assessment of the adaptive behavior score.

### Statistics

The level of significance was set at P<0.05. A comparison between the two independent groups was performed using Mann-Whitney’s U test. The Chi-square test was used to evaluate the significance of categorical variables. Correlation analysis was conducted using Spearman’s rank correlation coefficient test. Analyses were performed using GraphPad Prism version 6 software (GraphPad software, San Diego, USA). The difference of slopes between the groups was tested by the significance of the interaction term in multiple regression analysis. The association between the annual decrement of the social age and biomarkers with adjustment for age was examined by multiple regression analysis. Analyses were performed using SPSS for Windows version 23 software (IBM Japan Ltd., Tokyo, Japan).

## Results

**Fig 1:**
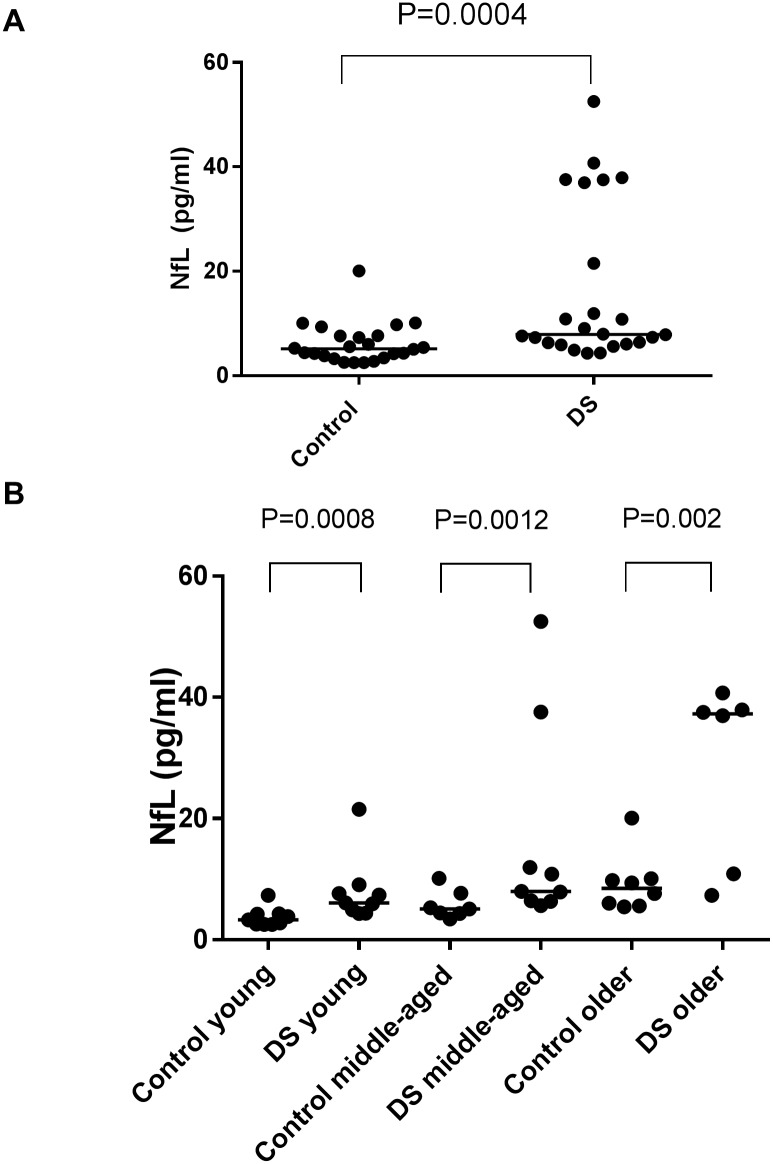
(A) Scatter plot of the NfL level in plasma in the control (n = 24) and DS (n = 24) groups. Bars indicate median values. Levels of NfL in the DS group were significantly higher than those in the control group (P = 0.0004). (B) Scatter plot of the NfL level in plasma in the young (aged 14–25 years; n = 9 in the control group and 9 in the DS group), middle-aged (aged 26–42 years; n = 7 in the control group and 9 in the DS group), and older (older than 43 years; n = 8 in the control group and 6 in the DS group) patients. Bars indicate median values. Levels of NfL in the DS group were significantly higher than those of the control group in all age groups (P = 0.0008 in the young, P = 0.0012 in the middle-aged, and 0.002 in the older patients).

**Fig 2:**
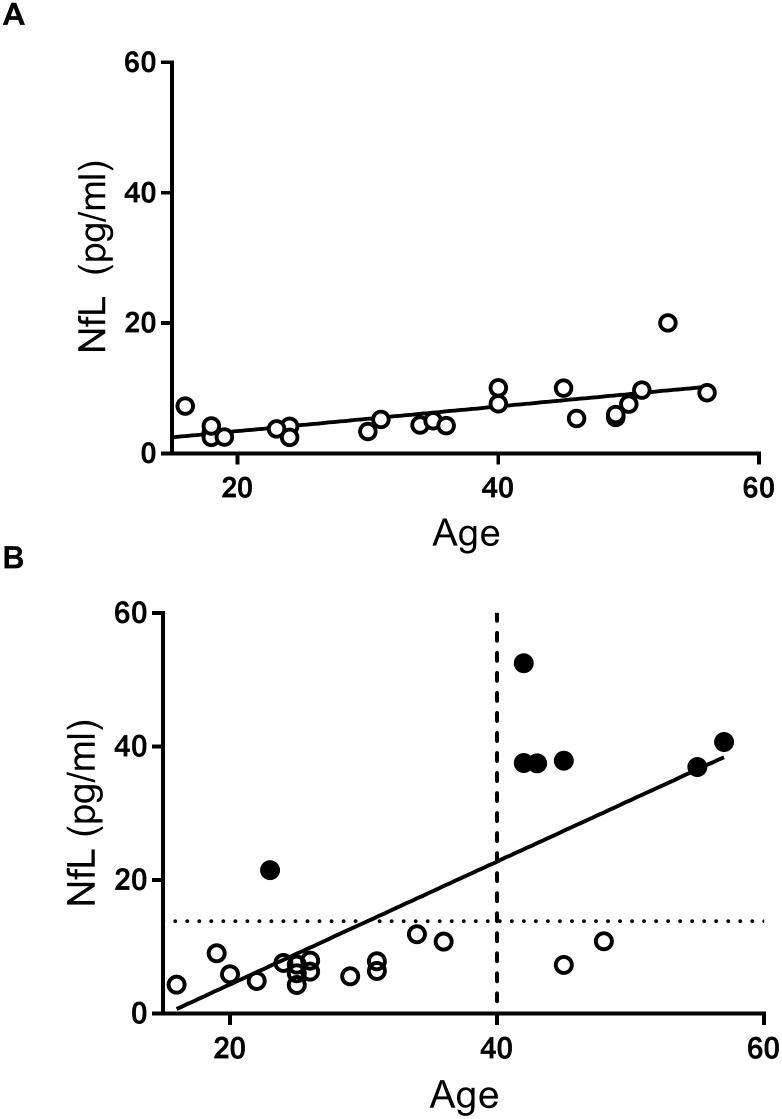
(A) Correlation between plasma NfL level and age in the control group. They showed a significant correlation. The P-value obtained from Spearman’s rank correlation coefficient test was <0.0001. (B) Correlation between NfL levels and ages in the DS group. A significant positive correlation was noted (P<0.0004). Filled circles (black) indicate the DS cases with NfL levels exceeding the cut-off value (13.872 pg/mL indicated by a dotted horizontal line), estimated from the control group. The dashed vertical line indicates the position at 40 years of age.

The demographic characteristics, cognitive assessments, comorbid diseases or condition and concentration of plasma NfL of participants are summarized in Tables 1 and 2. There was no DS patient with a history of head injury within the last three months. We found no significant difference in age (P = 0.858) or sex (P = 1.000) between the DS and control groups. As shown in Fig 1, levels of plasma NfL were significantly higher in the DS compared with the control group (P = 0.0004). When the participants were categorized by age into young (aged 14–25 years), middle-aged (aged 26–42 years), and older cases (over 43 years), the significant elevation of plasma NfL levels in the DS group was preserved in each age group. There was a significant correlation between age and level of plasma NfL in both the DS and control groups (Fig 2A and 2B), while the slope of the age-related change was steeper in the DS compared with the control group (the interaction term between the age and group was significant: P<0.001). When we set the cut-off value at 13.872 pg/mL based on the mean value and standard deviation (SD) in the control group (i.e., two SD above the mean value), the DS group contained seven patients with abnormally elevated plasma NfL according to this cut-off value (Table 2, Fig 2B). Six of these seven cases were over the age of 40. Regarding comorbidities, DS patients with epilepsy were older and had higher plasma NfL levels than those without epilepsy; however, these trends did not reach significance (P = 0.1097 and P = 0.0553, respectively). In the control group, plasma NfL levels were not significantly different between participants with and without epilepsy (P>0.999).

**Table 1:** Concentrations of plasma NfL in the control group.

Case	Sex	Age (years)	Comorbid disease or condition	NfL (pg/mL)
1	M	14	Epilepsy	2.766
2	F	18	Epilepsy	7.313
3	M	18	Epilepsy	3.257
4	F	18	None	2.531
5	F	18	None	4.259
6	F	19	None	2.551
7	M	23	Anxiety neurosis	3.813
8	F	24	Anxiety neurosis	4.230
9	F	24	None	2.528
10	M	30	Lumbar radiculopathy	3.426
11	F	31	None	5.280
12	F	34	None	4.422
13	M	35	None	5.092
14	M	36	None	4.308
15	M	40	None	7.677
16	F	40	Cavernous angioma	10.135
17	M	45	Diabetes mellitus	10.052
18	F	46	Depression	5.433
19	M	49	None	5.590
20	F	49	None	6.046
21	M	50	None	7.611
22	F	51	None	9.768
23	F	53	Epilepsy	20.096
24	M	56	None	9.372
	M:F 11:13	Mean±SD 34.1±13.6		Mean±SD 6.148±3.862

**Table 2:** Cognitive assessments and concentrations of plasma NfL in patients with DS.

Case	Sex	Age (years)	Comorbid disease or condition	DSQIID (total score)	Social Age (years)	%ΔSocial Age	NfL (pg/mL)
1*	M	16	None	10	4.00	N/A	4.398
2*	F	19	None	15	4.50	11.1%	9.092
3	F	20	None	0	5.42	N/A	5.935
4*	F	22	Hypothyroidism	0	7.42	-1.1%	4.921
5	F	23	None	5	5.33	N/A	21.532
6*	F	24	None	4	10.00	N/A	7.618
7*	F	25	None	0	7.00	N/A	6.069
8*	M	25	None	14	N/A	N/A	4.328
9*	M	25	Epilepsy	2	7.08	N/A	7.386
10*	F	26	None	1	4.17	N/A	7.954
11*	M	26	Epilepsy	0	6.50	7.7%	6.319
12*	M	29	None	36	4.67	-5.4%	5.650
13*	F	31	None	15	N/A	N/A	7.890
14*	M	31	Sleep apnea	2	2.33	3.6%	6.443
15	F	34	None	13	N/A	N/A	11.936
16*	M	36	Epilepsy	2	8.75	14.3%	10.807
17*	F	42	Epilepsy	17	4.17	-54.0%	52.535
18	M	42	Epilepsy	1	3.58	N/A	37.593
19*	M	43	None	10	6.08	-21.9%	37.546
20*	F	45	Epilepsy	13	1.92	-47.8%	37.930
21*	F	45	None	22	8.33	-1.0%	7.348
22*	M	48	Hyperuricemia	33	4.42	-22.6%	10.854
23*	F	55	Epilepsy	10	2.42	-62.1%	36.972
24*	M	57	None	22	4.08	-78.0%	40.749
	M:F 11:13	Mean±SD 32.9±11.6		Median 10	Mean±SD 4.86±2.40		Mean±SD 16.241±15.009

Note: We previously reported plasma levels of total tau [11] and phosphorylated tau [10] in cases with an asterisk. The DSQIID total score indicates the sum total of the scores from parts 2 and 3, which provides maximal sensitivity (0.92) and optimal specificity (0.97) for dementia screening in DS patients [19]. Social ages were estimated using the social maturity scale revised (S-M). Although data were calculated as units of years and months [18], we recalculated those into units of years for statistical analysis. Social ages in this figure are presented as units of years. There were seven patients with plasma NfL levels exceeding the cut-off value (13.872 pg/mL: two SD above the mean of those in the control group) (indicated by gray shading). N/A: not available. Comorbid disease or condition: treatment or diagnosis of sleep apnea, epilepsy, uricemia, hypothyroidism, and head injury in the last three months. No patient with multiple comorbid diseases was observed in the DS group.

**Fig 3:**
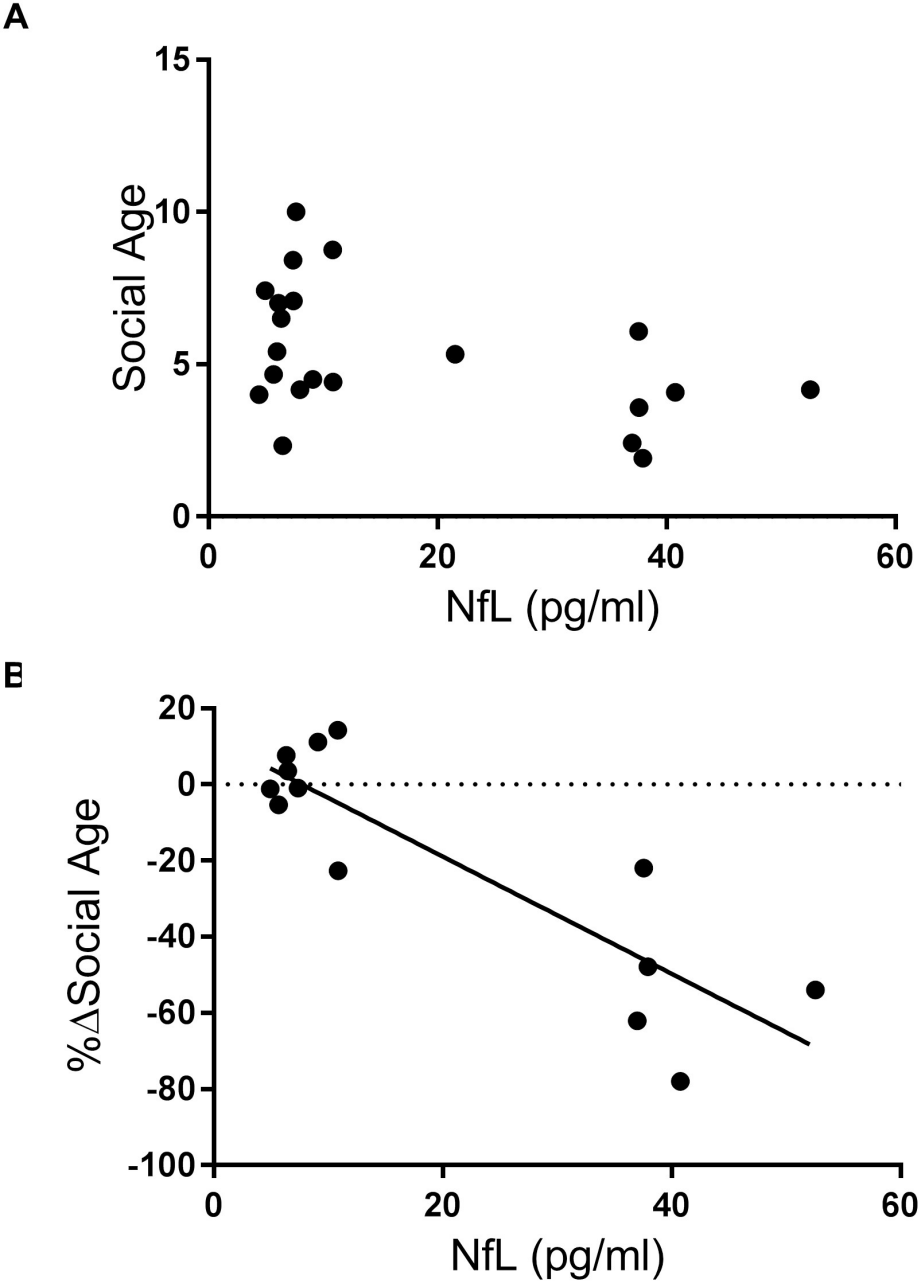
Correlation between NfL levels and social ages (A) and between NfL levels and %ΔSocial Age (B) in the DS group. The Spearman’s rank correlation coefficient test showed a non-significant association (P = 0.0887) between NfL and social ages (A), while a significant correlation was observed between NfL and %ΔSocial Age (P = 0.0001) (B).

The relationship between plasma NfL levels and social ages in patients with DS did not reach significance (P = 0.0887), but there was a significant negative correlation between %ΔSocial Age scores and plasma NfL levels (P = 0.0001) (Fig 3A and 3B). This negative correlation remained significant after age-adjustment (P = 0.005). Regarding the data on levels of plasma t-tau and p-tau that we have reported elsewhere from the same cohort, significant positive correlations between plasma levels of NfL and those of both t-tau and p-tau were observed (S1 Fig) [10, 11]. Furthermore, plasma levels of p-tau also showed a significant negative correlation with %ΔSocial Age, similar to the relationship between plasma NfL vs. %ΔSocial Age, although plasma t-tau failed to show a significant correlation in this analysis (S2 Fig). However, this significant correlation disappeared after age-adjustment (P = 0.080). Goodness of fit in the comparison of %Δsocial age was the most favorable for plasma NfL among these three biomarkers.

## Discussion

The present study showed that plasma levels of NfL were increased with age both in the control and DS groups. This age-dependent elevation of plasma and/or serum NfL in the healthy control group is in agreement with several previous reports [15] [16] [17]. The mechanism of this positive association between NfL concentration in body fluids and normal aging has not been clearly established, although it is considered to result from age-related subclinical axonal degeneration [20]. Consistent with a previous study on plasma NfL in DS [13], an age-dependent increase of plasma NfL levels was noted in the current study. Furthermore, the slope of this age-dependent elevation of plasma NfL was steeper in the DS than the control group. Consequently, plasma NfL levels were significantly higher in the DS group. Considering that an elevation of plasma NfL in patients with AD was repeatedly confirmed in several studies [15] [17] [21], our results can be interpreted as being a consequence of AD pathology in DS patients. This is supported by the fact that the majority of the seven patients with abnormally high levels of plasma NfL were over forty years old, which is around the age when tau pathology starts in DS individuals [3].

On the other hand, the significant elevation of plasma NfL in the DS group was observed not only in middle and older-age individuals, but also even in those under 26 years old. Actually, one of the seven with abnormal titers of plasma NfL was aged 23. Considering the fact that AD pathology has been rarely observed in such young individuals [3], elevated plasma NfL in young individuals with DS might result from DS-specific neurodegenerative factors distinct from AD pathology, such as disrupted proteostasis by trisomy 21 [22].

Plasma levels of NfL were negatively correlated with annual changes in the social age of the DS group even after age-adjustment. This result is consistent with a previous report that individuals with DS that converted to dementia had higher plasma NfL values compared with non-converters [13]. Although plasma levels of p-tau were also associated with this annual adaptive behavioral decrement, p-tau was not an independent predictor of dementia after age-adjustment. Social Ages of the S-M scale has not been established as a diagnostic or surrogate assessment for dementia in DS patients. However, we believe that the method is reasonable for our purpose, considering the following three facts: 1) there are no standard screening or diagnostic tools regarding how dementia should be detected in DS patients although its clinical diagnosis is quite robust [5, 23–25]; 2) similar adaptive behavior scores including those using the Vineland Social Maturity Scale have been reported to be affected by aging in DS patients [26–33]; 3) the S-M scale is a semi-quantitative assessment standardized with substantial data on typically developed controls speaking the language of our cohort. A recent study in rodent models of AD showed that NfL levels in CSF and plasma were attenuated by treatment with a β-secretase 1 inhibitor [34]. In fact, the normalization of serum/plasma NfL as a response to treatment has already been demonstrated in clinical trials of multiple sclerosis [35] [36]. These findings provide evidence to support the suggestion that plasma NfL is a potential biomarker to assess disease progression and monitor treatment effects in individuals receiving disease-modifying therapies in future clinical trials.

We acknowledge that the small sample size was a major limitation of this study and therefore, future studies to validate our findings are needed. Furthermore, the short follow-up period may have weakened the statistical power to detect the association between functional decline and the biomarkers. As we did not measure NfL in the follow-up plasma samples, longitudinal changes of plasma NfL in the DS population remain unknown. In the future, case-control as well as longitudinal studies involving sufficient numbers of participants with a longer follow-up period and repeated measurements of plasma NfL will be necessary to confirm our findings and promote the clinical application of the biomarker-supported diagnosis of dementia in DS patients.

## Conclusions

The study showed age-dependent increases in plasma NfL in both DS and control groups. This age-dependent elevation was steeper in the DS than the control group. Elevated plasma NfL was associated with decreased adaptive behavior scores one year later, providing evidence to support the suggestion that plasma NfL has the potential to serve as an objective biomarker to predict dementia in adult DS patients. The small sample size and the short follow-up period were major limitations of this study. Future case-control as well as longitudinal studies involving sufficient numbers of participants with a longer follow-up period and repeated measurements of plasma NfL are needed to confirm those findings.

## Supporting information

S1 Fig Correlation between plasma NfL level and tau-related biomarkers in the DS group. Plasma NfL levels were significantly correlated with t-tau (P = 0.0005)(A) and p-tau (P = 0.0085)(B). (Statistical analyses were conducted using Spearman’s rank correlation coefficient test).
(TIF)

S2 Fig Correlation between %ΔSocial Age and tau-related biomarkers in the DS group. %ΔSocial Age decreased with t-tau, but the relationship did not reach significance (P = 0.0741)(A), while there was a significant negative correlation between %ΔSocial Age and p-tau (P = 0.0048)(B). (Statistical analyses were conducted using Spearman’s rank correlation coefficient test).
(TIF)
