# Supplementary figures and images for: Plasma neurofilament light chain: A potential prognostic biomarker of dementia in adult Down syndrome patients
Source: PLoS One. 2019 Apr 5;14(4):e0211575. doi: 10.1371/journal.pone.0211575 (PMC6450630; doi:10.1371/journal.pone.0211575)

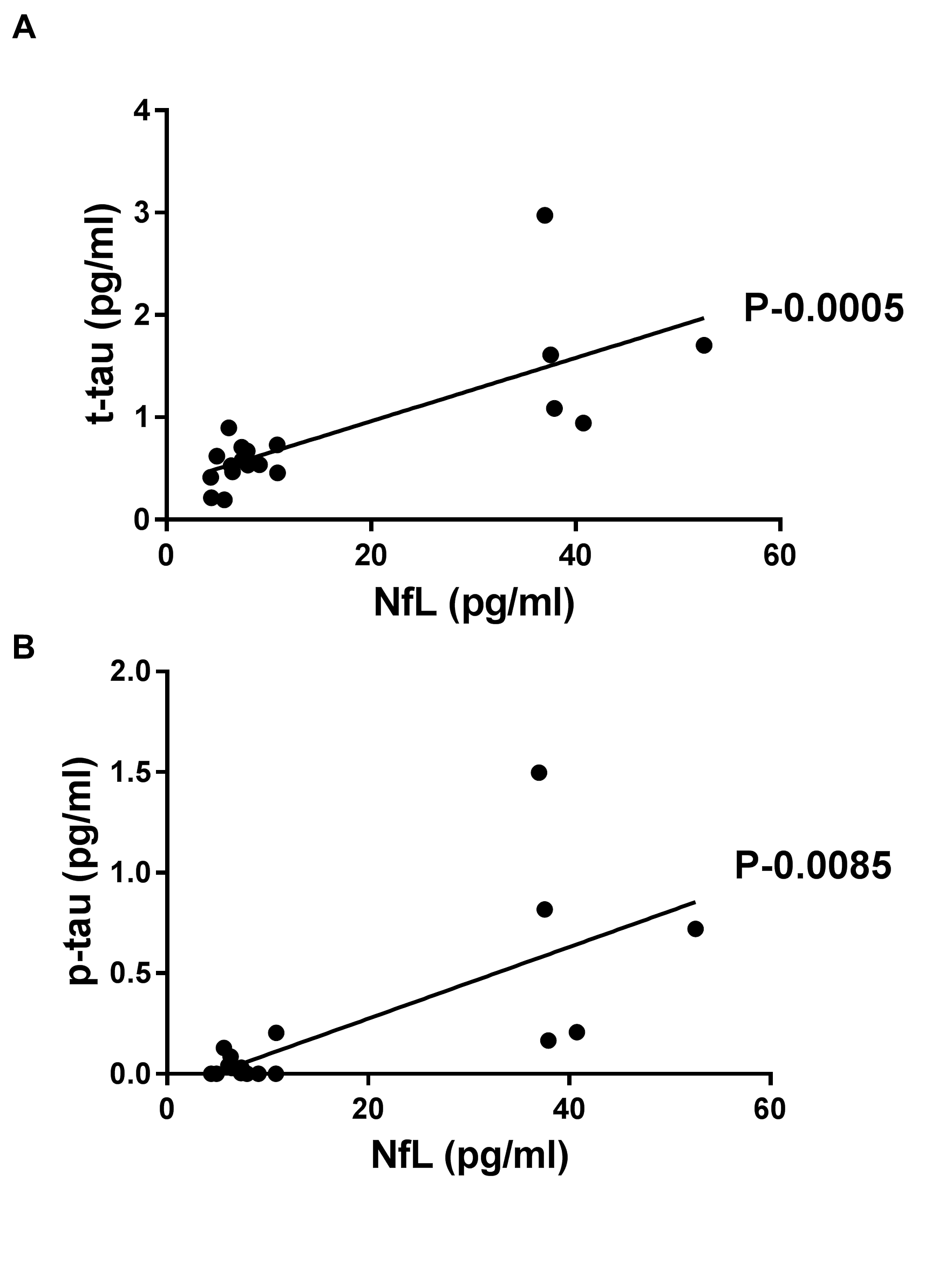

Supplement: S1 Fig — Plasma NfL levels were significantly correlated with t-tau (P = 0.0005)(A) and p-tau (P = 0.0085)(B). (Statistical analyses were conducted using Spearman’s rank correlation coefficient test). (TIF) [file pone.0211575.s001.TIF]

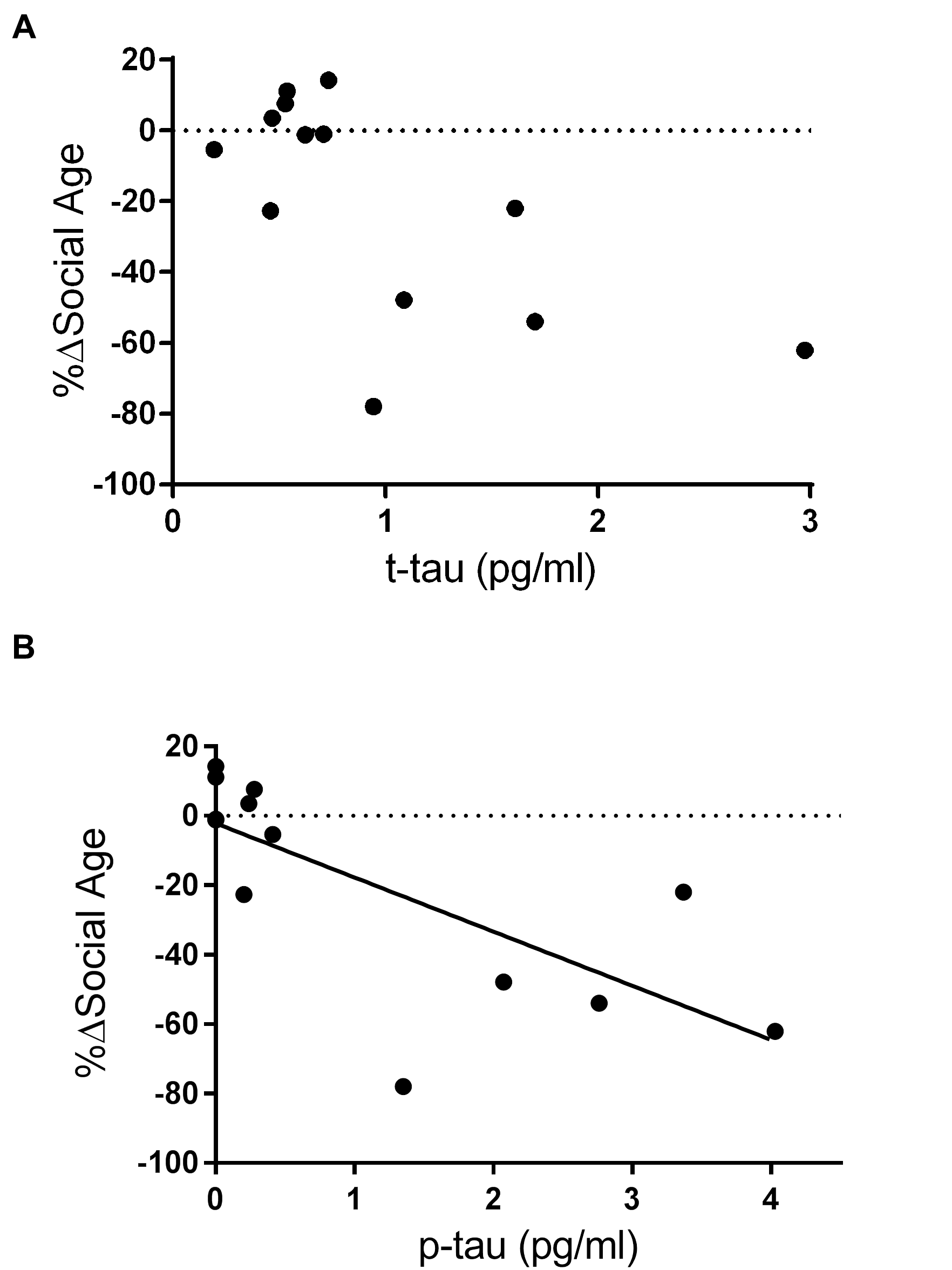

Supplement: S2 Fig — %ΔSocial Age decreased with t-tau, but the relationship did not reach significance (P = 0.0741)(A), while there was a significant negative correlation between %ΔSocial Age and p-tau (P = 0.0048)(B). (Statistical analyses were conducted using Spearman’s rank correlation coefficient test). (TIF) [file pone.0211575.s002.TIF]
